# Tuning the interfacial spin-orbit coupling with ferroelectricity

**DOI:** 10.1038/s41467-020-16401-7

**Published:** 2020-05-26

**Authors:** Mei Fang, Yanmei Wang, Hui Wang, Yusheng Hou, Eric Vetter, Yunfang Kou, Wenting Yang, Lifeng Yin, Zhu Xiao, Zhou Li, Lu Jiang, Ho Nyung Lee, Shufeng Zhang, Ruqian Wu, Xiaoshan Xu, Dali Sun, Jian Shen

**Affiliations:** 1 State Key Laboratory of Surface Physics and Department of Physics, Fudan University, 200433 Shanghai, China; 2 Hunan Key Laboratory of Super Microstructure and Ultrafast Process, School of Physics and Electronics, Central South University, 410083 Changsha, Hunan China; 3 Department of Physics and Astronomy, University of California, Irvine, CA 92697 USA; 4 Department of Physics, North Carolina State University, Raleigh, NC 27695 USA; 5 Department of Materials Science and Engineering, North Carolina State University, Raleigh, NC 27695 USA; 6 Institute for Nanoelectronics Devices and Quantum Computing, Fudan University, 200433 Shanghai, China; 7 Collaborative Innovation Center of Advanced Microstructures, 210093 Nanjing, China; 8 School of Materials Science and Engineering, Central South University, 410083 Changsha, Hunan China; 9 Materials Science and Technology Division, Oak Ridge National Laboratory, Oak Ridge, TN 37831 USA; 10 Department of Physics, University of Arizona, Tucson, AZ 85721 USA; 11 Department of Physics and Astronomy, Nebraska Center for Materials and Nanoscience, University of Nebraska, Lincoln, NE 68588 USA; 12 Organic and Carbon Electronics Lab (ORaCEL), North Carolina State University, Raleigh, NC 27695 USA

**Keywords:** Electronic devices, Spintronics

## Abstract

Detection and manipulation of spin current lie in the core of spintronics. Here we report an active control of a net spin Hall angle, *θ*_SHE_(net), in Pt at an interface with a ferroelectric material PZT (PbZr_0.2_Ti_0.8_O_3_), using its ferroelectric polarization. The spin Hall angle in the ultra-thin Pt layer is measured using the inverse spin Hall effect with a pulsed tunneling current from a ferromagnetic La_0.67_Sr_0.33_MnO_3_ electrode. The effect of the ferroelectric polarization on *θ*_SHE_(net) is enhanced when the thickness of the Pt layer is reduced. When the Pt layer is thinner than 6 nm, switching the ferroelectric polarization even changes the sign of *θ*_SHE_(net). This is attributed to the reversed polarity of the spin Hall angle in the 1^st^-layer Pt at the PZT/Pt interface when the ferroelectric polarization is inverted, as supported by the first-principles calculations. These findings suggest a route for designing future energy efficient spin-orbitronic devices using ferroelectric control.

## Abstract (web summary)

The spin Hall angle (SHA) is a measure of the efficiency for converting a charge to a spin current is still challenging to tune in situ. Here, the authors demonstrate by introducing a ferroelectric (FE) material in a ferromagnetic/heavy metal stack the SHA can be voltage controled via the polarization of the FE layer.

## Introduction

Spin-orbitronics, an emerging field that studies the coupling between electrons’ spin and orbital degrees of freedom, has recently drawn great attention as it offers a new route for the next-generation spintronic devices^1–4^. Instead of utilizing the spin-polarized charge current found in typical spintronics devices, spin orbitronics manipulates the degree of charge-spin conversion (i.e., spin Hall effect, or inverse spin Hall effect) by tuning the spin-orbit interaction^5,6^. The efficiency of this conversion is described by the spin Hall angle, *θ*_SHE_. While passive control of *θ*_SHE_ in metals (e.g., Pt, W, and Ta) has been routinely achieved on a variety of fronts by varying the resistivity^7–10^, alloying^11–15^, oxygen-level^16,17^, and concentration of Pt atoms in polymers through chemical synthesis^18^, active control of *θ*_SHE_ in metals is still challenging. The electric-field-induced enhancement of *θ*_SHE_ via an intervalley transition in a doped GaAs system^19^ appears to be a promising approach in terms of actively controlling *θ*_SHE_. However, further increase of *θ*_SHE_ is limited by the possible strength of electric field provided by an external voltage bias.

Ferroelectric materials, on the other hand, can generate a strong local electric field from their spontaneous electric polarization (e.g., ~80 µCcm^−2^ in PbZr_0.2_Ti_0.8_O_3_ or PZT)^20^ whose direction can be actively controled. The electric tunability of ferroelectric materials has been incorporated into a variety of spintronic devices since it potentially offers a non-volatile control of the spin degree of freedom for future spin memory and logic devices^3,4,21–24^.

**Fig. 1:**
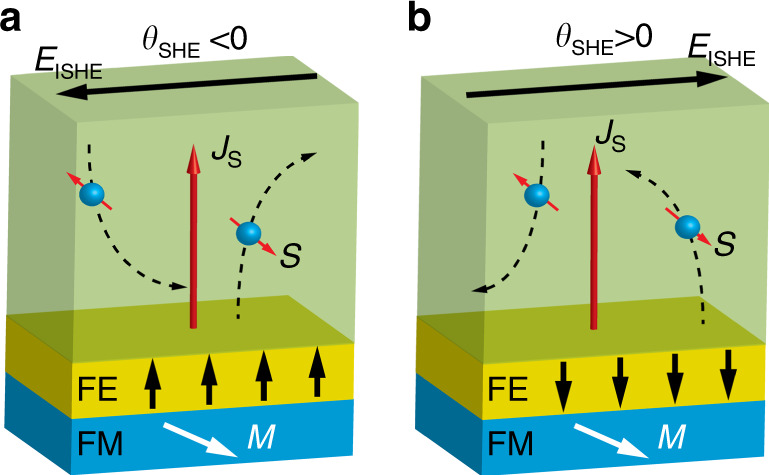
Ferroelectric control of spin Hall angle. The spin-polarized electrons from the FM electrode tunneled into the metal are deflected sideways due to the ISHE. **M**, **J**_**S**_, **S**, ***E***_ISHE_, and *θ*_SHE_ denote the magnetization of the FM electrode, the tunneled spin-polarized current, the spin-polarization vector, the generated electric field, and the spin Hall angle, respectively. The built-in electric field from the FE surface dramatically influences the electronic structure of the Pt layer at the interface and reverses the sign of the spin Hall angle, from which the detected ISHE voltage can be reversed based on the polarization state of PZT film: **a** the up and **b** the down polarization states, as the arrows shown in the figures.

In this work, we integrate ferroelectricity into a spin-orbitronic device in order to manipulate the spin-charge conversion in Pt, a material that is often used as a spin detector due to its large spin Hall angle. This new tunability can be understood as a result of energy-landscape modification of the interfacial Pt layer due to the large electric polarization of PZT (Fig. 1), which leads to either a shift of the density of states or emergence of the Rashba-splitting states, resulting in the change of polarity of the spin-charge conversion in the Pt layer.

## Results

### Characterizations of polarization reversal in PZT

**Fig. 2:**
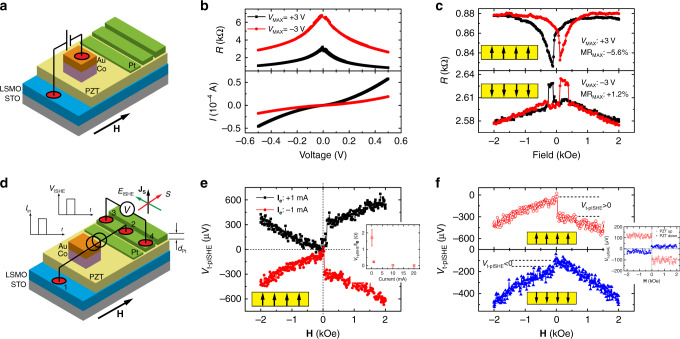
Ferroelectric control of spin transport in MFTJ and ISHE devices. **a** Schematic structure of the LSMO/PZT/Co (MFTJ-type device). **b** I(V) and R(V) curves of the MFTJ-type device with the polarization of PZT switched by V_MAX_ = +3.0 V (black squares) and −3.0 V (red circles), corresponding to PZT polarized up and down, respectively. **c** TMR loops of MFTJs measured at V_MR_ = −0.5 V for PZT polarized up (top panel) and down (bottom panel), showing a negative and positive TMR response, respectively. The inserted diagram in each panel stands for the ferroelectric states of the PZT layer. **d** Schematic illustrations of tunneling pulsed ISHE measurements in the ISHE-type device on the same substrate with the MFTJ-type device. The injected pulsed tunneling current (**I**_**e**_) generates a flow of pulsed spin current (**J**_**S**_) in the Pt metal, which produces a transverse pulsed ISHE voltage (V_t-pISHE_). **e** typical measured *V*_t-pISHE_(**H**) plots at **I**_**e**_  = ±1 mA in one ISHE-type device with as-grown PZT film (5 nm thickness, polarization pointing up), respectively. The magnetic field is swept from negative to positive field. The inset shows the measured *V*_t-pISHE_/**I**_**e**_ as a function of current. The error bars represent the standard deviation obtained from three different measurements. **f** The raw *V*_t-pISHE_(**H**) response (**I**_**e**_ = −1 mA) at two polarization states of PZT layer. The inset figure shows the response after subtracting the symmetric AMR response. The reversed voltage jumps around **H** = 40 Oe indicates a reversed ISHE response in the Pt metal. All the measurements were taken at 10 K, and the current density is ~10 Acm^−2^.

A schematic diagram of the multiferroic tunneling junction (MFTJ)-type device (Device A-MFTJ) is shown in Fig. 2a. La_0.67_Sr_0.33_MnO_3_ (LSMO) and Co films with distinct coercive fields are used as ferromagnetic electrodes; the tunneling barrier consists of a ferroelectric PZT layer epitaxially grown on LSMO^22^. For PZT with a thickness of 5 nm, the polarization of the as-grown ferroelectric (FE) state points up (see Supplementary Note 1). The polarization-down state of a PZT film can be achieved by applying a voltage bias, e.g., using a piezoelectric force microscope (PFM) probe (see Supplementary Fig. 1b–c). In the geometry of the MFTJ-type device, an external ramping DC voltage bias, V_MAX,_ applied between the LSMO and the top Co electrodes, is used to switch and reset the polarization state of the PZT^22^. Fig. 2b shows measured R–V and I–V characteristics of the MFTJ-type device after applying V_MAX_ = +3.0 V (PZT poled up) and −3.0 V (PZT poled down), respectively. A clear and reversible change of the device resistance between two different ramping voltages is attributed to a typical tunnel-electroresistance (TER) response in the FE-based MTJ device^25^. This is related to the change of the height of the PZT tunnel barrier caused by the FE polarization reversal^26–28^. The observation of a TER response provides direct evidence of the electric-field control of the FE polarization by applying the ramping DC voltage. Figure 2c shows the measured tunneling magnetoresistance (TMR) response at the same measuring voltage, V_MR_ ( | V_MR_ | < | V_MAX_ | ) of the Device A-MFTJ. The TMR magnitude is defined as TMR = (R_AP_ − R_P_)/R_P_ × 100%, where R_p_ and R_AP_ represent the device resistances in the parallel and antiparallel magnetic configurations, respectively. The reversed sign of the TMR response at each opposite FE polarization state is consistent with previous reports^25^, although the origin of the TMR reversal is still under debate. One plausible explanation is that the movement of Ti atoms in the Ti–O plane of the PZT layer (i.e., toward and away from the interfacial Co layer) would dramatically influence the magnetic moment of Co due to the hybridization or proximity effect^21^. Consequently, the degree of spin polarization at the PZT/Co interface may be reversed. However, in such a MFTJ configuration containing two ferromagnetic electrodes, it is generally difficult to distinguish whether the reversed spin polarization occurs at the interface of LSMO/PZT or PZT/Co^3,21,29,30^.

The TMR(V_MR_) profile follows the gradual switching of the PZT polarization from pointing fully up (at V_MAX_ ≥ +2.0 V) to pointing fully down (at V_MAX_ = −3.0 V)^25^. An asymmetrical plot of TMR vs V_MR_ is observed. This may originate from the different work functions of the FM electrodes and the substrate-related built-in field (PZT polarization prefers to point up in the as-grown state). The decay of TMR values at higher V_MR_ has been explained by the magnon excitation mechanism^31^, i.e., injected hot electrons at a higher voltage excite magnons and randomize the majority and minority states leading to a decrease of the spin polarization at the surface of the FM electrode. From the TMR(V_MR_) profile and Julliere’s model^32^, the voltage dependence of the spin polarization of the two FM electrodes (i.e., P_S_(V)_LSMO_ and P_S_(V)_Co_) at each FE polarization state can be derived (see Supplementary Note 2). This offers a guideline for correlating the effect of spin polarization with the observed tunneling pulsed inverse spin Hall effect (t-pISHE) response, as discussed in the next section.

### Ferroelectric control of net spin Hall angle

The reversal of the TMR response in Fig. 2c demonstrates the high-quality nature of the PZT tunneling barrier, which exhibits reversible FE polarization after the deposition of the top metal electrode. It also confirms that the current tunneled from LSMO through PZT into the top electrode is spin polarized. When the top electrode is replaced with spin-orbit coupled materials, such as Pt, the spin-polarized current along the out-of-plane direction in Pt is expected to generate a transverse charge current according to the inverse spin Hall effect (ISHE). Below we demonstrate that this spin-charge conversion through ISHE in the Pt layer can be controled by switching the FE polarization of the PZT layer, with an effect large enough to reverse the polarity.

Figure 2d shows the schematic structure of the ISHE-type device (Device A-ISHE) on the same PZT substrate where the MFTJ-type device was measured. Here, the spin current (**J**_**S**_) across the junction and the Pt electrode along the out-of-plane direction is carried by the pulsed spin-polarized charge current (**J**_**e**_) that flows between leads #1 and #2 by tunneling through the PZT barrier; ${\mathrm{J}}_{\mathrm{e}} = {\mathrm{I}}_{\mathrm{e}}/{\mathrm{A}}$, where I_e_ is the applied charge current and A is contact area of lead #2. The relation between the charge and spin current is ${\mathrm{J}}_{\mathrm{S}} = {\mathrm{P}}_{\mathrm{S}}({\mathrm{V}}_{\mathrm{e}})_{{\mathrm{LSMO}}}{\mathrm{J}}_{\mathrm{e}}$, where P_S_(V_e_) is the finite-bias spin polarization of LSMO derived from the MFTJ-type device (see Supplementary Note 2); ${\mathrm{V}}_{\mathrm{e}} = {\mathrm{I}}_{\mathrm{e}}{\mathrm{R}}$ where R is the device resistance of the ISHE-type junction. A pulsed transverse charge current (${\mathbf{J}}_{\mathbf{c}} = \sigma {\boldsymbol{E}}_{{\mathrm{t}} - {\mathrm{pISHE}}}$) will be generated between leads #3 and #4 via the ISHE in the Pt strip, which can be expressed by^33^:

1 $${\mathbf{J}}_{\mathbf{c}} = \sigma {\boldsymbol{E}}_{{\mathrm{t}} - {\mathrm{pISHE}}} = \theta _{{\mathrm{SHE}}}({\mathrm{net}}){\mathbf{J}}_{\mathbf{s}} \times {\mathbf{S}}$$

where **J**_**c**_, **σ**, ***E***_t-pISHE_, and **S** are the generated transverse charge current, conductivity of Pt, transverse t-pISHE field, and spin polarization direction vector of the injected spin current, respectively; here *θ*_SHE_(net) is an overall efficiency of the spin-charge conversion for the ISHE device as shown in Fig. 2d, which has the contributions from the Pt/PZT interface and the bulk Pt.

The advantages of using t-pISHE technique are: (i) At a high voltage bias, the generated ISHE response is attributed to hot spin-polarized electrons in contrast to the electrons at the Fermi surface of the FM electrode using the spin-pumping technique^34^; (ii) Owing to the short pulse duration time of electrical excitation (delta mode), the t-pISHE technique effectively eliminates spurious thermoelectric effects, such as the Seebeck effect, spin-dependent Seebeck effect, and anomalous Nernst effect, etc^3,18,35–38^; As a result, *θ*_SHE_(net) decreases rapidly when the current increases, suggesting a decay of spin polarization at finite bias (see the inset in Fig. 2e and Supplementary Note 3)^39^, in contrast to the quadratic increase of voltage ($\propto {\mathrm{I}}_{\mathrm{e}}^2$) due to the joule heating; (iii) The effect of anisotropic magnetoresistance (AMR), anomalous Hall effect, and planar Hall effect (PHE) signals stemming from the bottom LSMO electrode^39,40^ would be greatly suppressed due to the electrical isolation induced by the tunnel barrier between the Pt and the LSMO electrode.

Figure 2e shows the typical measured t-pISHE signals at two pulsed charge currents (I_e_ **=** ±1 mA) on the as-grown PZT substrate (i.e., polarization up). The positive (negative) polarity of the applied charge current represents the spin-polarized electrons being injected into (from) the LSMO bottom electrode. An abrupt voltage jump in the vicinity of zero magnetic field is observed while sweeping the magnetic field (**H**) along the **S**-direction. The switching field of ~40 Oe coincides with the coercive field of the LSMO film (see Supplementary Fig. 1d). The voltage changes in the vicinity of the switching magnetic field are defined as $V_{{\mathrm{t}} - {\mathrm{pISHE}}} \equiv \Delta V = V_{ - H} - V_{ + H}$, which is −0.23 ± 0.03 mV and +0.22 ± 0.03 mV at I_e_ **=** +1 mA and I_e_ **=** −1 mA, respectively. The measured voltage magnitude at I_e_ = +1 mA (−1 mA) is slightly different, in accordance with the asymmetric bias dependence of the electron spin polarization into (from) the LSMO electrode. The inset shows the measured transverse t-pISHE resistance $( {| {V_{{\mathrm{t}} - {\mathrm{pISHE}}}} |/{\mathrm{I}}_{\mathrm{e}}} )$ as a function of applied charge current (see Supplementary Fig. 3a–d and i, the t-pISHE original data detected using different current I_e_ for Device A-ISHE). The significant drop at higher magnitudes of current density (requiring a higher applied voltage bias) agrees with the decay of voltage-dependent spin polarization of the LSMO electrode as demonstrated in Device A-MFTJ^25^. The magnetic-field-dependent hysteretic background is attributed to the ‘residual’ contributions of AMR, PHE, and/or tunneling anisotropic magnetoresistance^39^ occurring in the LSMO film when a charge current passes through the film (see Supplementary Note 4). The inset of Fig. 2f presents the extracted actual $V_{{\mathrm{t}} - {\mathrm{pISHE}}}$(*H*) loops after this background is subtracted from the data (see Supplementary Note 6).

In the Device A-ISHE, the switching of the PZT polarization is achieved by applying a higher pulsed charge current (up to ±30 mA) using a high voltage bias, with a similar purpose as the ramping voltage used in the Device A-MFTJ device. The t-pISHE measurement was conducted using a “delta” mode at a lower current value, i.e. alternating positive (+1 mA) and negative pulse current (−1 mA) to eliminate the thermal-related artifacts, immediately after the high current excitation. Remarkably, for the same pulse current I_e_ = −1 mA, we found that the polarity of measured *V*_t-pISHE_ is inverted between the PZT polarization up $( {V_{{\mathrm{t}} - {\mathrm{pISHE}}\left( \uparrow \right)} = 0.22 \pm 0.03\,{\mathrm{mV}}} )$ and polarization-down $( {V_{{\mathrm{t}} - {\mathrm{pISHE}}\left( \downarrow \right)} = - 0.04 \pm 0.03\,{\mathrm{mV}}} )$.

**Fig. 3:**
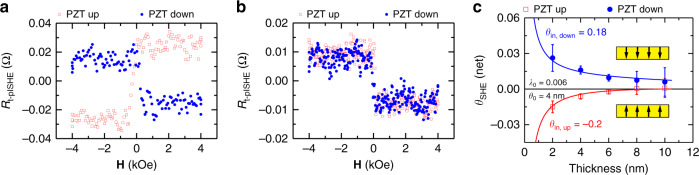
Pt-thickness-dependent t-pISHE response at the two ferroelectric polarization states of PZT. **a**, **b** t-pISHE response in 6 nm and 8 nm Pt (Devices B series) at two PZT polarization states of pointing up (red empty squares) and down (blue solid circles), respectively. **c** net spin Hall angle $( {\theta _{{\mathrm{SHE}}}({\mathrm{net}}) = \frac{{V_{{\mathrm{t}} - {\mathrm{pISHE}}}\sigma }}{{{\mathrm{J}}_{\mathrm{e}}{\mathrm{P}}_{{\mathrm{LSMO}}}{\mathrm{L}}}}} )$ as a function of Pt thickness (Devices C series). The red (blue) solid lines through the data points are fits to *θ*_SHE_(net) using Eq. (2) based on two PZT polarization states. The respective spin Hall angles extracted from the fits are denoted. Fitting parameters: *θ*_0_ = 0.006, *λ*_in_ = 0.2 nm*, λ*_0_ = 4 nm. The error bars represent the standard deviation obtained from three different measurements.

Figure 3 presents the Pt-thickness-dependence of t-pISHE signal in ISHE-type devices prepared from the same batch using the PLD technique (Device B and Device C, see Methods). For a 6-nm-thick Pt strip (Device B-6 nm, Fig. 3a), $V_{{\mathrm{t}} - {\mathrm{pISHE}}}$ switches its polarity from negative to positive when the PZT polarization switches from the up to the down direction, whereas for the 8-nm-thick Pt strip, $V_{{\mathrm{t}} - {\mathrm{pISHE}}}$ exhibits a positive value regardless of the polarization direction of the PZT layer (Device B-8 nm, Fig. 3b). Figure 3c summarizes the measured $\theta _{{\mathrm{SHE}}}({\mathrm{net}}) \equiv \frac{{\sigma V_{{\mathrm{t}} - {\mathrm{pISHE}}}}}{{{\mathrm{J}}_{\mathrm{e}}{\mathrm{P}}_{\mathrm{S}}{\mathrm{L}}}}$ (see Supplementary Note 7) as a function of Pt thickness ($d_{{\mathrm{Pt}}} = 2 - 10\;{\mathrm{nm}}$ from Device C) at both PZT polarization states, where L is the length of the Pt stripe and *σ* is the bulk conductivity of Pt. The magnitude of *θ*_SHE_(net) decays when *d*_Pt_ increases, since the out-of-plane spin current vanishes in the part of Pt far away from the interface. Remarkably, when the Pt thickness is low (<6 nm), we found that the sign of *θ*_SHE_(net) is inverted when the FE polarization of PZT is reversed: $\theta _{{\mathrm{SHE}}}^ \downarrow$(net) is positive when the FE polarization points to downward direction, whereas $\theta _{{\mathrm{SHE}}}^ \uparrow$(net) is negative at the same thickness if the FE polarization is switched to an upward direction. In comparison, *θ*_SHE_ of a Pt films deposited directly on the ferromagnetic layer is always positive, which has been treated as a standard value for the spin-to-charge convertor as reported elsewhere^41,42^. Therefore, the reversed sign of *θ*_SHE_(net) at lower Pt thicknesses demonstrates the active electric-field control of the spin-charge conversion vias ISHE with ferroelectricity.

## Discussion

Thermally-related effects (e.g., ordinary Seebeck, spin-Seebeck effect, anomalous Nernst effect, planar Nernst effect, etc.) in our ISHE-type device have been suppressed through the use of the pulsed tunneling ISHE method^18^ with the delta model. The residual contribution of AMR and TAMR effects from the LSMO film can be separated from the actual t-pISHE signal due to their hysteretic response by sweeping a magnetic field (see Supplementary Note 4). AHE and PHE are also greatly suppressed by inserting the insulating PZT barrier between the Pt and LSMO films^39^. Proximity effects that are usually observed in the Pt/ferromagnet bilayer system can also be ruled out by inserting the 5 nm nonmagnetic PZT insulating layer^43^.

The effect of PZT FE polarization on the spin polarization at the PZT/LSMO interface is unlikely to cause the observed *θ*_SHE_(net) reversal. First, *θ*_SHE_(net) has a significant dependence on the thickness of the Pt layer, which is not obviously related to the properties of the PZT/LSMO interface. Second, the optimally-doped LSMO used in this work has a robust magnetic order, corresponding to its high Curie temperature (>300 K) and insensitivity of magnetism to magnetoelectric effects^44,45^.

Using a control experiment, we studied the effect of PZT polarization reversal on the PZT/LSMO interfacial spin polarization. By inserting a thin Cu layer (two monolayers) between the PZT and the Pt layer, i.e., constructing a LSMO/PZT/Cu/Pt device, we carried out the same t-pISHE measurement (Device A-Cu, see Supplementary Note 5). We found that the ISHE polarity remains the same after the PZT polarization reversal, suggesting a minimal spin-polarization change at the PZT/LSMO interface after the PZT polarization reversal. This result actually demonstrates that, in a LSMO/PZT/Co MFTJ, the Co/PZT interface is more likely to be responsible for the FE-controled TMR response in (Fig. 2c) than it is for the PZT/LSMO interface, which has been under debate for years^21,29^.

After ruling out all these scenarios that may affect *θ*_SHE_(net), it is clear that the sign reversal of *θ*_SHE_(net) is determined by both the polarization state of PZT and the thickness of Pt layers (Fig. 3c). Here we introduce a phenomenological model about the existence of an additional interfacial spin Hall angle (*θ*_in_) in the ultrathin Pt layer at the PZT/Pt interface that dominates the sign reversal. With this, the Pt-thickness dependence of *θ*_SHE_(net) can be written as^18,46^ (see Supplementary Note 7):

2 $$\theta _{{\mathrm{SHE}}}({\mathrm{net}}) = \frac{{\theta _{{\mathrm{in}}}^{ \uparrow ( \downarrow )}\lambda _{{\mathrm{in}}} + \theta _0\lambda _0{\mathrm{tanh}}(\frac{{d_{{\mathrm{Pt}}}}}{{2\Delta \lambda _0}})}}{{d_{{\mathrm{Pt}}}}}$$

where *λ*_in_ = 0.1–0.2 nm is the inelastic electron scattering length for electrons at the interface of PZT/Pt, *θ*_0_ and *λ*_0_ are the bulk spin Hall angle and spin diffusion length of the Pt film taken from the literature, respectively^8,41^. For simplicity, *λ*_in_ is assumed to be unchanged with respect to the PZT polarization. For the best fitting, it is found that *θ*_0_ = 0.006, *λ*_in_ = 0.2 nm*, λ*_0_ = 4 nm, and the interfacial spin Hall angle, *θ*_in_ is −0.20 for PZT polarization up and +0.18 for PZT polarization-down states, respectively. At smaller Pt thickness, the contribution of interfacial *θ*_in_ is much larger than that of bulk *θ*_0_, leading to the sign reversal of *θ*_SHE_(net). At larger Pt thickness, the bulk *θ*_0_ governs the overall spin-to-charge conversion, and therefore no sign change of *θ*_SHE_(net) was observed. Hence, the most striking experimental observation here is the active control of interfacial spin Hall angle *θ*_in_ in the ultrathin Pt film, up to a sign change, by switching the electric polarization of the PZT layer using an external electric field. This is in contrast to tuning the spin Hall angle in metals using passive approaches like varying conductivity^7–9^ or alloying^11–15^.

**Fig. 4:**
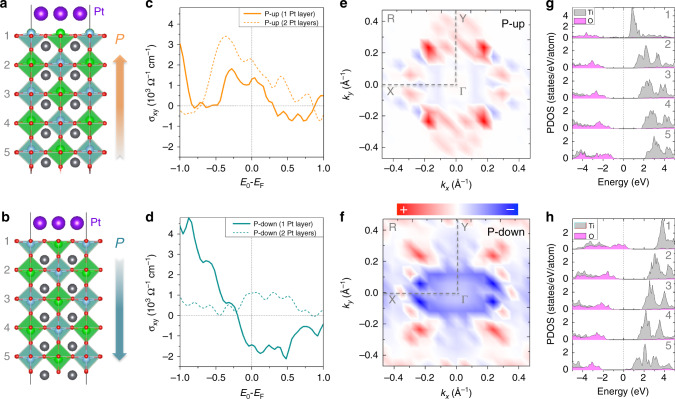
DFT calculations. **a**, **b** Atomic model for a Pt adlayer on the PZT substrate with upward and downward ferroelectric polarizations out of the surface. **c**, **d** Calculated spin Hall conductivity *σ*_xy_ of 1 Pt/PZT and 2 Pt/PZT. **e**, **f** Distributions of the Berry curvature in the two-dimensional Brillouin zone. Red and blue represent positive and negative contributions of Berry curvature, respectively. **g**, **h** Calculated layer projected density of states of Ti and O atoms in the PZT substrate. The vertical dashed line at E = 0 represents the position of the Fermi level. The numbers indicate the corresponding layers of Ti and O atoms counting from the interface. Pink and gray denote O-2p and Ti-3d contributions, respectively.

In principle, the reversal of the PZT polarization originates from the movement of Ti/Zr atoms, accompanied with the atomic lattice displacement at the PZT/Pt interface. The strong electric field generated by the PZT film may also introduce screening charges at the interface. Both structural and electrostatic effects may noticeably alter the electronic structure of the Pt atoms at the interface, resulting in the sign change of the interfacial spin Hall angle. This conjecture is supported by density functional theory (DFT) calculations using the Vienna Ab-initio Simulation Package (VASP)^47^. As sketched in Fig. 4a–b, a 2 × 2 supercell for the PZT substrate and a 3 × 3 supercell for Pt interfacial layers are used in accordance with a lattice mismatch of ~4% between the PZT and Pt structure. The Ti–O (or Zr-O) termination^21,48^ was assumed at the PZT/Pt interface where the switch of relative positions of the Ti and O planes is applied to simulate the PZT electric polarization. Spin Hall conductivity (SHC) *σ*_xy_ was directly calculated from the Berry curvature to compare with the experimental results.

In Fig. 4c–d, *σ*_xy_ of the monolayer and double Pt layer on top of PZT is plotted against $E_{\mathrm{0}} - E_{\mathrm{F}}$ for the upward and downward FE polarizations, respectively, where *E*_0_ is the highest occupied energy level. As shown in Fig. 4c, curves of *σ*_xy_ of the monolayer and double Pt layers exhibit a similar trend in a broad range of *E*_0_ for the upward FE polarization. Around the Fermi level, *E*_0_ = *E*_F_, *σ*_xy_ values are +2280 Ω^−1^cm^−1^ for monolayer Pt layer and +1309 Ω^−1^cm^−1^ for the double Pt layer, respectively. When the PZT polarization is switched to the downward direction (Fig. 4d), the magnitude of the *σ*_xy_ of the double Pt layer significantly drops but the sign remains positive. Nevertheless, *σ*_xy_ of the monolayer Pt layer becomes negative at $E_{\mathrm{0}} - E_{\mathrm{F}} \,> - \!0.2\,{\mathrm{eV}}$ and has a negative value of −1428 Ω^−1^cm^−1^ at $E_{\mathrm{0}} = E_{\mathrm{F}}$. Although it is still difficult to completely separate the interface and bulk contributions to *σ*_xy_ in such an ultrathin thickness regime, it is clear that the interfacial component accounts for the sign reversal of *σ*_xy_ when the PZT polarization is inverted.

To further elucidate the origin of the sign change of the interfacial *σ*_xy_, we show the distribution of Berry curvature in the two-dimensional Brillouin zone (2DBZ) of the monolayer Pt layer on the PZT. As shown in Fig. 4e, the positive *σ*_xy_ for the upward electric polarization mainly results from four red spots between the **Γ** and **R** points. When the polarization is switched down, the presence of a blue region appearing around the center of 2DBZ (Fig. 4f) suggests that the sign change of the interfacial *σ*_xy_ stems from the change of states around the **Γ** point. The change of the electronic structure of PZT/Pt caused by the polarization reversal is shown in Fig. 4g–h in the form of projected density of states (PDOS) of Ti, O atoms at different layers in PZT. Driven by the strong internal electric field, the band edges bend oppositely as we switch the direction of polarization of PZT. When the ferroelectric polarization points in the upward direction, the conduction bands of Ti atoms at the interface shift to the Fermi level and interact with Pt atoms around *E*_F_. In contrast, the valence bands of interfacial O atoms hybridize with Pt atoms around *E*_F_ when the polarization is reversed. Therefore, Pt atoms sense two distinct interfaces when the direction of polarization is inverted, which leads to the reversible change of the interfacial spin Hall angle, particularly in the first Pt layer.

Although our DFT calculations suggest a significant change of *σ*_xy_, accompanied by a possible sign reversal in the interfacial Pt layer when the PZT polarization is reversed, the sign of the observed *θ*_SHE_ and the calculated *σ*_xy_, unfortunately, do not always agree, indicating the complexity of the phenomena beyond the proposed simple model here. Several missing components in the calculation may contribute to the imperfect agreement. For instance, any possible interdiffusion/mixing/redox state between PZT and Pt atoms may cause the complicated interfacial electronic structural changes and thus affect the *σ*_xy_ of Pt atoms dramatically. As demonstrated in Supplementary Note 8, when the Pt and O atoms are interchanged at the PZT/Pt interface, the calculated *σ*_xy_ of the monolayer Pt layer at the PZT up polarization can indeed be reversed. Furthermore, the electric field generated by the PZT affects the charge accumulation in the interfacial Pt layer and shifts *E*_0_ with respect to *E*_F_. Given the ultrasensitive *σ*_xy_ as a function of *E*_0_ around *E*_F_ shown in Fig. 4c–d, a modest shift of *E*_0_ combined with the structural reconstruction may lead to a substantial sign change of *σ*_xy_.

The Rashba effect on the spin-to-charge conversion at the PZT/Pt interface has also been investigated. Supplementary Note 9 show the DFT band structures for both upward and downward PZT polarization states. By projecting the bands to the monolayer Pt layer and to two spin channels, a pair of Rashba spin-splitting bands is observed in the case of the upward PZT polarization. The calculated Rashba parameter is $\alpha_{\mathrm{R}}^\uparrow=-152\,{\mathrm{meV}}\cdot\,$Å. In contrast, there is no Pt Rashba band when the PZT polarization switches to the downward direction. Assuming that the spin momentum time, τ, in the Pt layer is 5 fs and the thickness of the interface, *d*_in_, is 0.2 nm, the derived inverse Rashba–Edelstein effect (IREE) length, $\lambda _{{\mathrm{IREE}}} = \alpha _{\mathrm{R}}^ \uparrow \tau /\hbar$, is 0.02 nm and the additional contribution from the IREE to an effective spin Hall angle is $\theta _{{\mathrm{SHE}}}^{\mathrm{IREE}}$ = 2$\lambda _{{\mathrm{IREE}}}/d_{{\mathrm{in}}}$ = −0.18 for the upward polarization, in agreement with the estimated interfacial spin Hall angle in Eq. (2).

The present study provides a robust technique for the electrical control of spin-charge conversion in metals at interface with ferroelectrics, where the electric field is several orders of magnitude higher than that produced by conventional voltage bias^19^. This offers electric tunability of spin Hall effect not only in other metals (such as W, having the largest spin Hall angle^41^) but also in inorganic and even organic semiconductors^18^. The achievement of the reversed sign of spin Hall angle in the heavy element Pt, considered as the standard spin-current detector, suggests that electric field driven by ferroelectricity is a promising approach for manipulating spin-to-charge interconversion for future spin-torque based memory and logic devices. Our research paves the way for future studies of ferroelectricity functionalized spin-orbitronics.

## Methods

### Film depositions

A Pulsed Laser Deposition system was used to grow 40 nm LSMO and then 5 nm PZT films epitaxially on the surface of a SrTiO_3_ (STO) substrate. The topography and the ferroelectric properties of the LSMO/PZT films were characterized by a Veeco Dimension 3100 at room temperature with atomically flat surfaces and switchable electric polarization^22,49,50^. The magnetic properties of the LSMO films were measured by a Quantum Design Superconducting Quantum Interference Device (SQUID).

### Fabrications of multiferroic tunneling junctions (Device A-MFTJ)

Ten nanometer thick patterned Co electrodes and ~10 nm Au cap layers were deposited on top of LSMO/PZT films by thermal evaporation in a high vacuum chamber (base pressure of <1 × 10^−6^ Pa) using a shadow mask to fabricate junctions with an area of ~100 × 100 μm^2^.

### Fabrications of inverse spin Hall effect devices

The Pt stripes (width: 200 μm; length: 4 mm for Device A series and B series, and 2 mm for Device C series) were deposited on the top of LSMO/PZT by e-beam evaporation (e.g., Device A-ISHE on the same LSMO/PZT film for preparing the MFTJ device, and the thickness of Pt is 4 nm), or by PLD technique (Device B-ISHE, with 6-nm- and 8-nm-thick Pt, and Devices C-ISHE with a series of controled Pt thickness of 2, 4, 6, 8, and 10 nm on two different LSMO/PZT films, respectively. For the control device, LSMO/PZT/Cu/Pt device (Device A-Cu), two monolayers (0.7 nm) Cu is deposited on top of the same LSMO/PZT of device A using e-beam evaporation, followed by 4 nm Pt with the same dimensions as the Pt stripes as that in other ISHE devices. Silver paste and Au wires were used for electrical contact. Keithley 2400 source meter, 6221 current source, and 2182 A nanovoltmeter were used to detect the magnetoresistance and the pulsed ISHE voltage of the devices in the Physical Property Measurement System (PPMS, Quantum Design). Tunneling pulsed current (pulse duration length: 1 ms with the “delta” model) is used to measure ISHE response in Pt metals in order to suppress possible thermoelectric artifacts from the ferromagnetic films. All the transport measurements were taken at 10 K.

### Computer simulation

The generalized gradient approximation (GGA) was used for the description of the exchange-correlation interaction among electrons. We treated Pt-5d6s, Pb-6s6p, Zr-4d5s, Ti-3d4s, and O-2s2p as valence states and adopted the projector-augmented wave (PAW) pseudopotentials to represent the effect of their ionic cores^51,52^. The GGA+U method was used for the localized d-orbitals of Zr and Ti (U = 3.2 eV, J = 0.9 eV)^48^. We sampled the Brillouin zone (BZ) with the 5 × 5 × 1 and 17 × 17 × 1 **Γ**-centered Monkhorst-Pack **k**-meshs for structural relaxations and SHC calculations, respectively^53^. The energy cutoff for the plane-wave expansion was set to 400 eV. We fixed positions of atoms in the two bottom cubic cells and allowed other atoms to relax with a criterion that requires the atomic force on each atom smaller than 0.01 eV per Å and the energy convergence better than 10^−5^ eV. Spin-orbit coupling was included in the calculations of spin Hall conductivity.

## Supplementary information

Supplementary Information

## Data Availability

The data that support the findings of this study are available from the corresponding authors upon reasonable request.
