# Supplementary material for: Tuning the interfacial spin-orbit coupling with ferroelectricity
Source: Nat Commun. 2020 May 26;11:2627. doi: 10.1038/s41467-020-16401-7 (PMC7250895; doi:10.1038/s41467-020-16401-7)
Supplement: Supplementary file 1 — Supplementary Information [file 41467_2020_16401_MOESM1_ESM.pdf]

Supplementary Information

Tuning the Interfacial Spin-orbit Coupling with Ferroelectricity

Fang *et al.*

Content

Supplementary Note 1. The morphologies, ferroelectric and magnetic properties of the films and the devices

Supplementary Note 2. Estimation of spin polarization from the ferromagnetic electrode

Supplementary Note 3. Current density dependence of the t-pISHE signal

Supplementary Note 4. Magnetic field dependence of the t-pISHE signal

Supplementary Note 5. Control experiment: t-pISHE response in LSMO/PZT/Cu/Pt device

Supplementary Note 6. Data processing for the detected t-pISHE signal

Supplementary Note 7. Interfacial and bulk spin Hall angles

Supplementary Note 8. Interfacial complexity effects on spin Hall conductivity

Supplementary Note 9. The Rashba effect at the PZT/Pt interface

Supplementary Note 1. The morphologies, ferroelectric and magnetic properties of the films and the devices

40 nm LSMO and then 5 nm PZT films are epitaxially grown on an STO(100) substrate in high vacuum (base pressure < 10^{-6} Pa) by the pulsed laser deposition technique. The films are grown layer by layer and monitored by the reflection high energy electron diffraction (RHEED) with atomic flat surface, where the terrace of STO substrate can still be seen on the surface topography shown in Supplementary Figure 1a.[1] The ferroelectric properties of the PZT film are characterized by PFM: the as-grown films have an up-polarization; by applying a +5 V tip bias, the polarization of the film can be switched down, as shown in Supplementary Figure 1b. By gradually increasing the tip bias, the ferroelectric domains of PZT film can be partially switched by a +1.7 V tip bias (Supplementary Figure 1c). The magnetic properties of the ferromagnetic LSMO electrode are investigated by SQUID. From the magnetic hysteresis loops shown in Supplementary Figure 1d, the coercivity of the LSMO electrode is ~50 Oe.

The crosssectional image of LSMO/PZT/Pt ISHE-type devices is characterized using high-resolution TEM taken from High Angle Annular Dark Field (HAADF), as shown in Supplementary Figure 1e. The TEM sample is prepared via focus ion beam (FIB) milling. For device protection, ~10 nm Cr and ~1 μm thick Pt layers are deposited by sputtering before FIB milling. Each layer in the device is well-separated with sharp interface. The obtained thickness for each layer is in agreement with the thicknesses measured from RHEED and quartz crystal microbalance thickness monitor during the deposition.

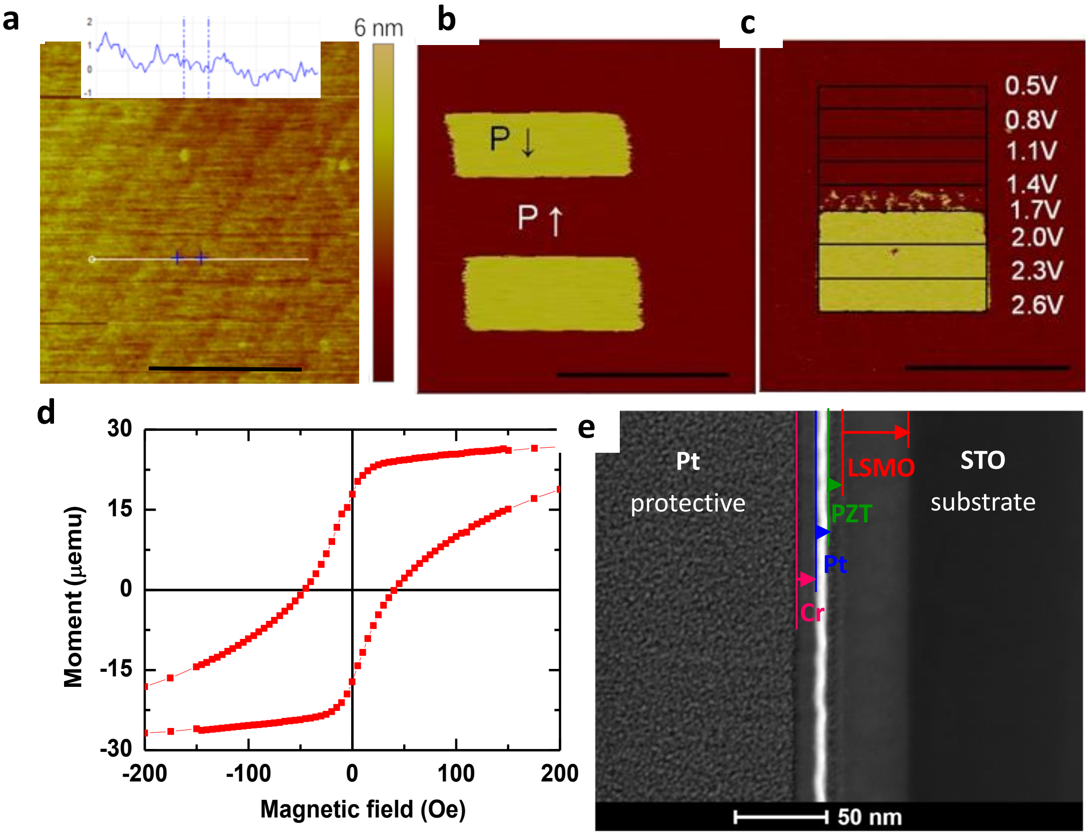

Supplementary Figure 1. Morphologies, ferroelectric and magnetic properties of the films and the devices: **a-c** The PFM images of the epitaxial grown LSMO/PZT film on the STO substrate. The scale bar is 1μm. The inset shows the atomically flat height profile along the white line for the prepared LSMO/PZT film. **d**. The magnetic hysteresis loops of the LSMO/PZT film. The measurements are taken at 10K. **e**. The crosssectional HAADF TEM image of the LSMO/PZT/Pt ISHE device.

Supplementary Note 2. Estimation of spin polarization from the ferromagnetic electrode

According to the magnon excitation mechanism, the hot electrons tunneling into/out of the ferromagnetic electrodes would reduce the spin polarization, leading to a decay of spin polarization with the bias and the corresponding dependence of TMR on measuring voltage.[2-4] The effect of the hot-electron injection on the interfacial spin polarization can be estimated from the dependence of TMR on V_{MR} using Julliére equation:[5]

$$\mathrm{TMR} = \frac{2P_1P_2}{1+P_1P_2} \times 100\% \quad (1)$$

where P_1 and P_2 stand for the effective spin polarization of LSMO and Co in the device, respectively. We fit the TMR vs. V_{MR} curves linearly at finite measurement voltage according to the sign of V_{MR} (the direction of the spin current) for V_{MAX}= +2.0 V, -2.0 V and -3.0 V, respectively (Supplementary Figure 2a). The decay rate of TMR (corresponding to the decay of spin polarization of electrons) for electrons tunneling into LSMO ($V_{MR} > 0$ region) in the device is much larger than that of Co ($V_{MR} < 0$ region), with a constant ratio of ~-2.5 shown in Supplementary Figure 2b. This decay of TMR is caused by the decay of spin polarization of the two ferromagnetic electrodes. Here we have proposed: (1) the decay of spin polarization caused by the hot electrons is a linear function of bias; (2) the decay rate of spin polarization for LSMO and Co is the same (*k*); and (3) the decay caused by hot holes (h) tunneling into the ferromagnetic electrode is ignorable compared with hot electrons (e).[3] The effective spin polarization of ferromagnetic electrodes at different measurement voltage can be described as:

$$P_{1,e}(V_{MR}) = P_1(0) - kV_{MR}\ ,\ P_{1,h}(V_{MR}) = P_1(0) \quad (2)$$

$$P_{2,e}(V_{MR}) = P_2(0) - kV_{MR}\ ,\ P_{2,h}(V_{MR}) = P_2(0) \quad (3)$$

where k is the decay rate of spin polarization of the LSMO and Co magnetic electrodes. For positive measurement voltage, the hot electrons (holes) tunneling into LSMO(Co) electrode, while for negative V_{MR}, the hot electrons (holes) tunneling into Co (LSMO) electrode in our MFTJs. Their products of effective spin polarizations are:

$$P_1P_2(V_{MR} > 0) = P_{1,h}(V_{MR})P_{2,e}(V_{MR}) = P_1(0)P_2(0) - P_1(0)kV_{MR} \quad (4)$$

$$P_1P_2(V_{MR} < 0) = P_{1,e}(V_{MR})P_{2,h}(V_{MR}) = P_1(0)P_2(0) - P_2(0)kV_{MR} \quad (5)$$

Meanwhile, the effective spin polarizations product can be deduced from the TMR according to the Julliére equation：

$$P_1(V_{MR})P_2(V_{MR}) = \frac{TMR}{2-TMR} \approx \frac{TMR}{2} \quad (6)$$

The decay rate of $P_1(V_{MR})P_2(V_{MR})$ is almost proportional to the decay rate of TMR at the region of $V_{MR}>0$ and $V_{MR}<0$, respectively. Therefore, $(P_1(0)k):(P_2(0)k) = -2.5$ according to the experimental fitting shown in Supplementary Figure 2b. We get $P_1(0) = -2.5P_2(0)$. The effective spin polarization of LSMO and Co can be estimated from the TMR values at different V_{MR}, as an example for V_{MAX}= +2.0 V presented in Supplementary Figure 2c.

Supplementary Figure 2d shows the effective spin polarizations of LSMO and Co electrodes under finite measuring voltage (i.e., ($P_1(0)$) and ($P_2(0)$), respectively) at different poling bias (V_{MAX}), indicating the effect of PZT polarization on the LSMO/PZT interfacial spin polarization. The effective spin polarization of LSMO changes from ~ 35% to ~ 20%, while for Co it changes from ~ -12% to ~ +8% after the PZT is switched from up to down state (corresponding to the V_{MAX} changes from positive to negative).

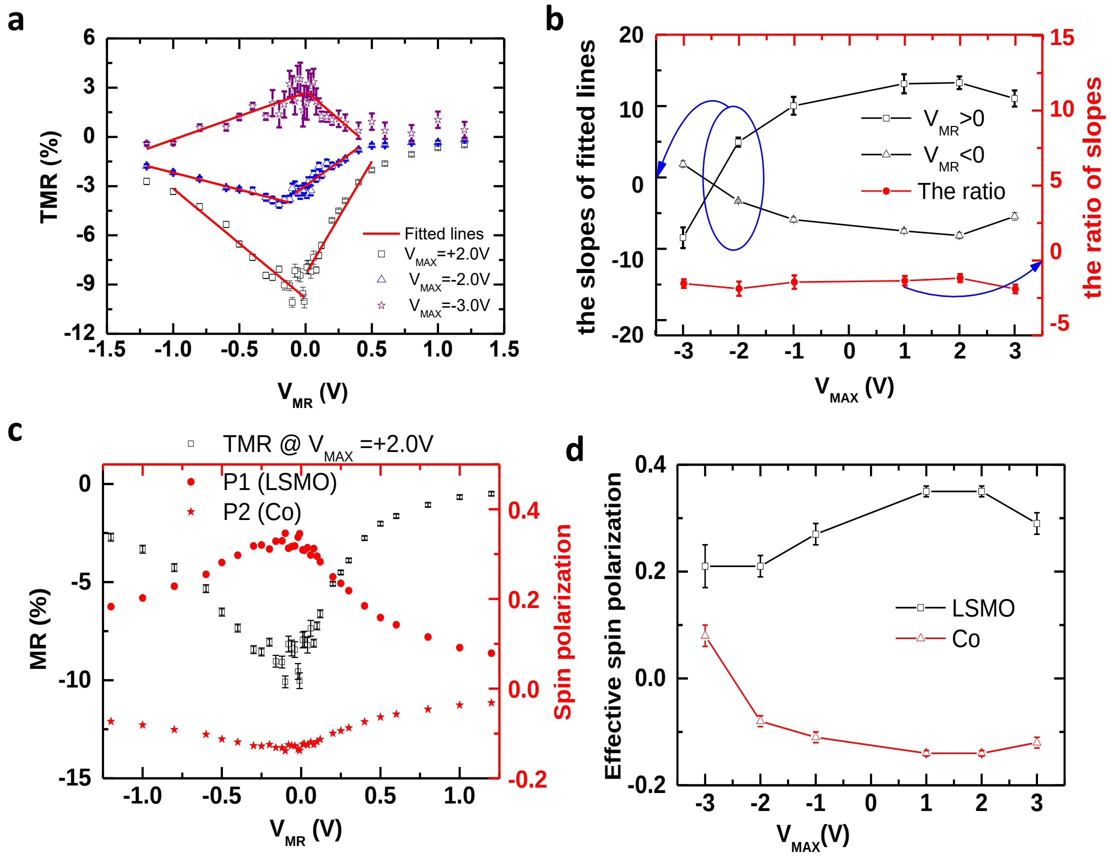

Supplementary Figure 2. Interface spin polarization: **a.** typical examples of fitted decay of TMR at finite bias according to magnon excitation mechanism. The values of poling bias for the TMR(V_{MR}) curves are V_{MAX} = +2.0 V, -2.0 V and -3.0 V. **b.** the decay rate (the slope of the fitted lines) and the ratio of the rates at positive and negative V_{MR}, tuned by the polling bias V_{MAX}. **c.** the effective spin polarizations of LSMO and Co estimated form the TMR values with dependence on measuring voltage at poling bias V_{MAX} = +2.0 V. **d.** The effect of V_{MAX} on the effective spin polarization of LSMO and Co at finite voltage. Error bars represent the standard deviation from three different detections.

Supplementary Note 3. Current density dependence of the t-pISHE signal

Supplementary Figure 3 shows the original transverse electrical voltage (i.e., ISHE response) as a function of magnetic field at different pulse current amplitudes in two devices (Device A-ISHE and Device C-ISHE), respectively. The as-grown PZT polarization is pointing upward in both devices. The Pt stripe in the Device A (Supplementary Figure 3a-d) was deposited using electron-beam evaporation with the deposition rate of 1 Åmin^{-1} for the first 1 nm and then 3 Åmin^{-1} for another 3 nm. For Device C (Supplementary Figure 3e-h), the Pt stripe was deposited by pulsed laser deposition with rate of 1 Åmin^{-1} and thickness of 4 nm.

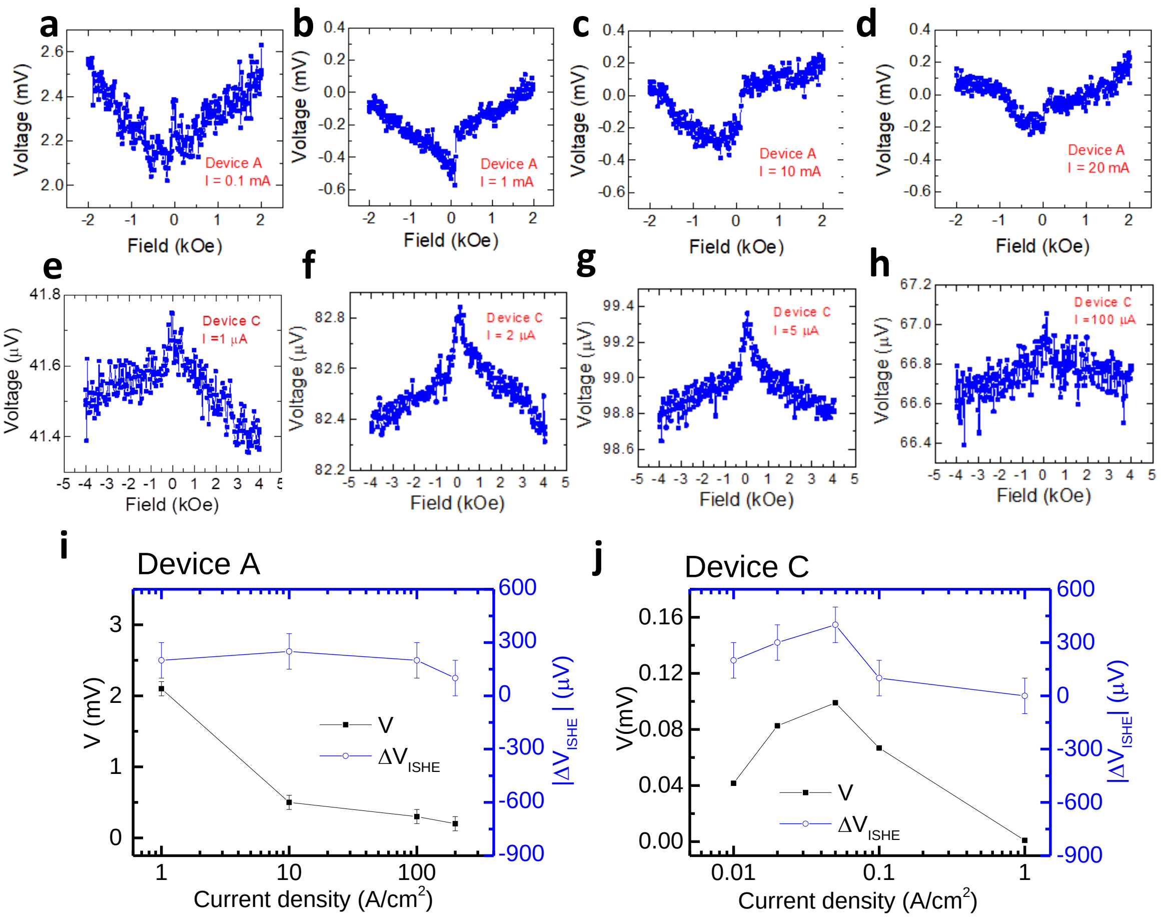

Supplementary Figure 3. Obtained t-pISHE (**H**) loops in Device A-ISHE and Device C-ISHE with different pulsed currents. **a.** 0.1 mA, **b.** 1 mA, **c.** 10 mA, **d.** 20 mA for Device A-ISHE; **e.** 1 μA, **f.** 2 μA, **g.** 5 μA, **h.** 100 μA for Device C-ISHE. **i** and **j**, the current dependence of the background voltage and ISHE voltage (ΔV_{ISHE}) for Device A and Device C, respectively. Error bars represent the standard deviation from three different detections.

The voltage jumps in the vicinity of the zero-field have been attributed to the spin-dependent ISHE signal, ΔV ($V_{\mathrm{t-pISHE}} \equiv \Delta V = V_{-H} - V_{+H}$). Presumably, the amplitude of ISHE voltage would be proportional to the injected tunneling current density ($\mathbf{J_c}$) as described in Eq.(1) in the paper: $\Delta V \sim \sigma E_{\mathrm{t-pISHE}} \propto \theta_{\mathrm{SHE}} \boldsymbol{J_s} \times \mathbf{S} = \theta_{\mathrm{SHE}} \mathrm{P(V)} \boldsymbol{J_c} \times \mathbf{S}$. This would lead to an increase of ISHE voltage when the applied pulse current increases. However, the spin polarization P(V) of the ferromagnetic electrode significantly decays at a high bias, as discussed in Supplementary Note 2. This results in the decrease of the ISHE voltage as shown in Supplementary Figure 3i-j. Furthermore, the current-induced phonon vibration may introduce more spin scattering and thereby reduces the net spin polarization as well as the ISHE voltage. It is noteworthy that the current dependence of t-pISHE signals strongly depends on the quality of the Pt stripes and LSMO/PZT thin film. As presented in Supplementary Figure 3i-j, while overall the tendency of the current-dependent t-pISHE signal are similar, two devices with the same thickness of Pt prepared by different techniques (E-beam evaporation for Device A and PLD for device C) exhibit different magnitude of ISHE voltage and current dependence. Since batch-to-batch sample variation cannot be avoided, we chose to fabricate both the MFTJ-type and the ISHE-type devices with different Pt thickness on the same PZT/LSMO for the direct comparison.

Supplementary Note 4. Magnetic field dependence of the t-pISHE signal

The t-pISHE(**H**) signal is recorded by sweeping the magnetic field along the in-plane direction. Supplementary Figure 4 shows the t-pISHE signal hysteresis loops detected at $I_C = \pm 1$ mA. The magnetization of the LSMO electrode is inverted at the coercive field of ~ ±50 Oe according to the magnetic hysteresis loop presented in Supplementary Figure 1d, consistent with the switching field of TMR response in MFTJs.[4] The switched magnetization reverses the spin polarization and results in a jump of t-pISHE voltage around this coercive field.

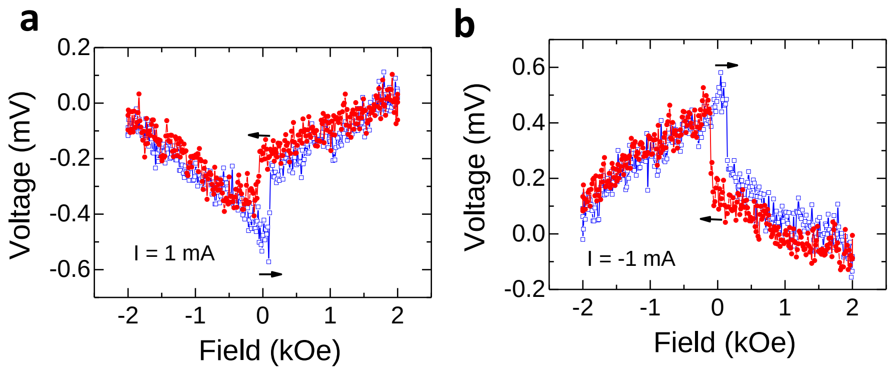

Supplementary Figure 4. Magnetic hysteresis of t-pISHE signal detected with pulsed curred of **a.** +1 mA and **b.** -1 mA. The current density is ~10 Acm^{-2}.

Supplementary Note 5. Control experiment: t-pISHE response in LSMO/PZT/Cu/Pt device

By inserting a thin Cu interlayer (2 monolayers, ~0.7 nm) between PZT and Pt film, we fabricate the control sample, i.e., LSMO/PZT/Cu/Pt device. Supplementary Figure 5 shows the measured t-pISHE signal in the LSMO/PZT/Cu/Pt device, compared with the LSMO/PZT/Pt device prepared on the same LSMO/PZT film. Whereas the t-pISHE signal $(V_{\mathrm{t-pISHE}})$ in the LSMO/PZT/Pt device switches its polarity when PZT polarization is inverted, there is no reversal of ISHE signal observed in the LSMO/PZT/Cu/Pt device $(V_{\mathrm{t-pISHE}} > 0)$. The absence of sign reversal in this control sample confirms the key role of the PZT/Pt interface for the ISHE response.

The amplitude of the applied current does not change the polarity of the t-pISHE response unless the PZT polarization is reversed (see Supplementary Note 3). Supplementary Figure 5g-i present the obtained t-pISHE signal from another batch of LSMO/PZT/Pt device measured at I = +2 μA (~ 0.02 $\mathrm{Acm^{-2}}$), which is three orders of magnitude smaller than that of the device A shown in the Supplementary Figure 5 a-c (measured at I = +1 mA, ~10 $\mathrm{Acm^{-2}}$). The t-pISHE still reverses with the swtich of FE polarization of PZT layer.

Yet, it is very challenging to measure the t-pISHE using exactly the same pulsed current for the two different LSMO/PZT/Pt and LSMO/PZT/Cu/Pt devices due to the changed device configuration. Supplementary Figure 6 shows a summary of $\Delta\mathrm{V}(V_{\mathrm{t-pISHE}})$ normalized by the pulsed currents for devices presented in our work with different FE polarization states of the PZT film. It is confirmed that there is no reverse of ISHE signal in Cu-based devices. For all the ISHE devise with Pt thickness ≤ 6 nm, the reversed ISHE signals have been clearly identified.

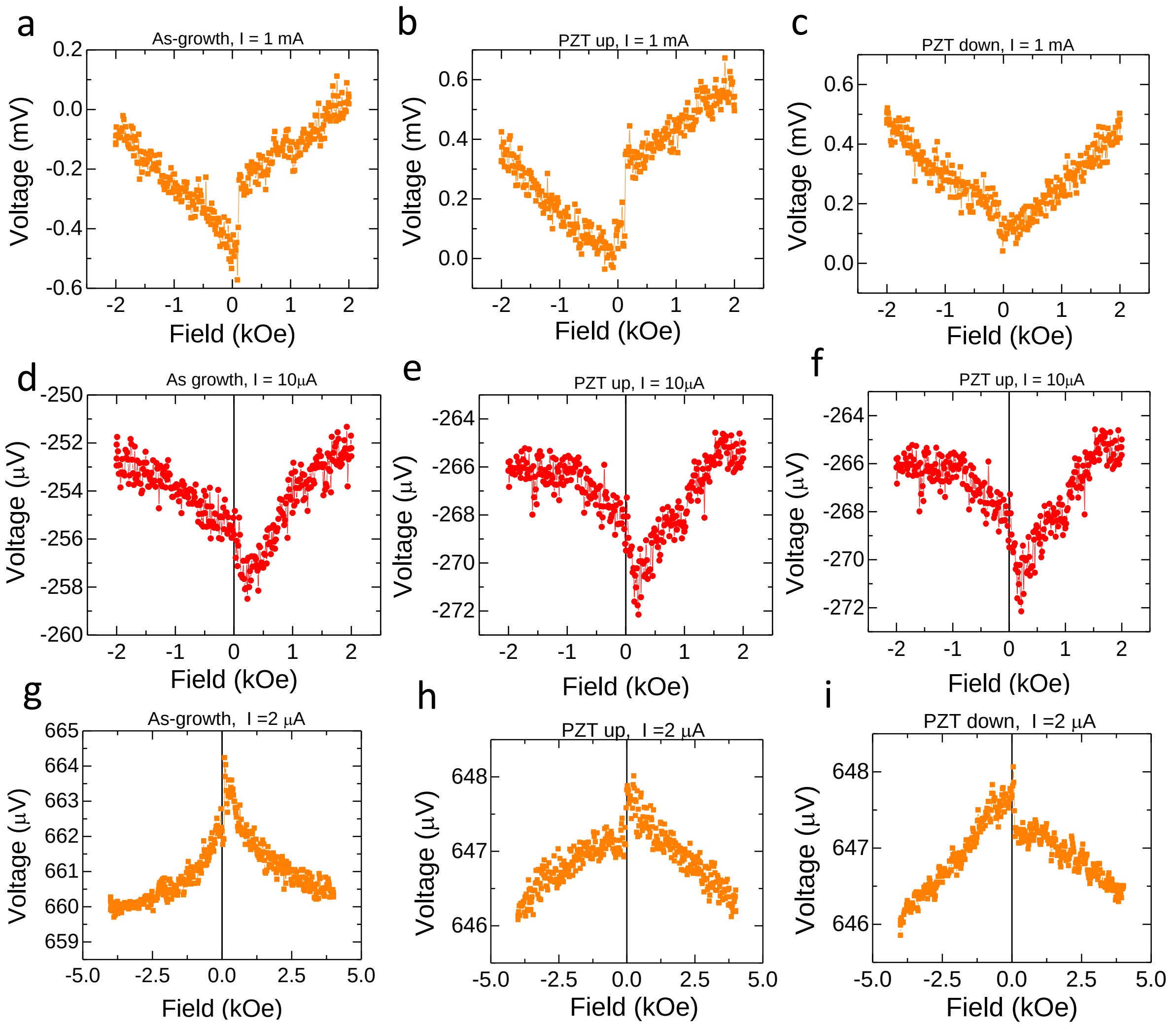

Supplementary Figure 5. t-pISHE signal of LSMO/PZT/Pt and LSMO/PZT/Cu/Pt devices. **a-c.** the t-pISHE response for Device-A-ISHE measured at I = +1 mA (~10 Acm^{-2}) with PZT states (**a.** as-growth, **b**. polarized down and **c.** polarized up). **d-f.** the t-pISHE response for Device A-Cu measured at I = +10 μA (~0.1 Acm^{-2}) with different PZT states (**d.** as-growth, **e.** polarized down and **f.** polarized up, respectively). **g-i.** the t-pISHE signal of another LMSO/PZT/Pt device (Device C) measure at I = +2 μA (~0.02 Acm^{-2}) with **g.** as-grown, **h.** polarized down and **i.** polarized up PZT. All the measurements were taken at 10K.

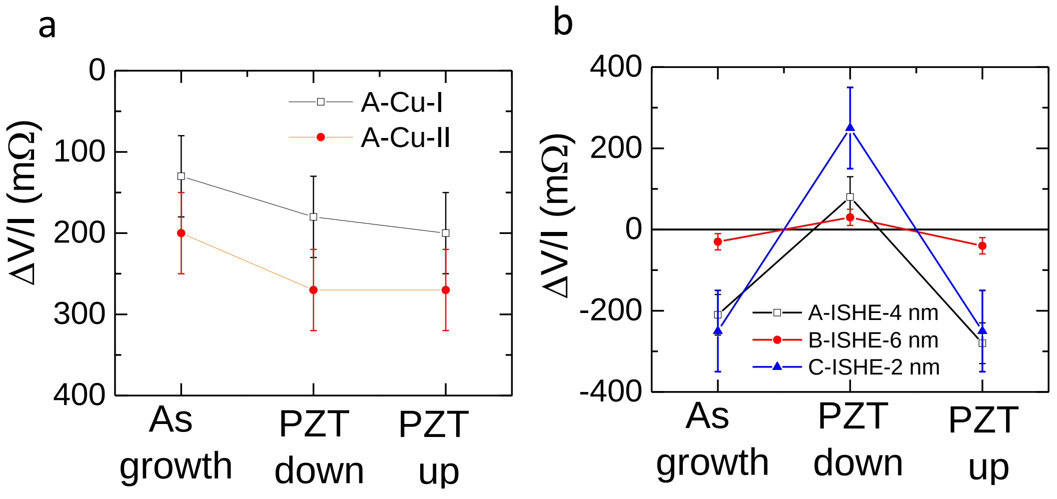

Supplementary Figure 6. The normalized t-pISHE signal for devices at different PZT polarziation states: **a.** Cu-based devices and **b.** ISHE devices with different Pt thickness. Error bars represent the standard deviation from three different detections.

Supplementary Note 6. Data processing for the detected t-ISHE signal

The as-detected ISHE data contains a field-dependent non-hysteresis background, which could be caused by the residual contribution of the anisotropy tunneling magnetoresistance, the anomalous and the planar Nernst effect in LSMO electrode.[6] We subtract this background to obtain the t-pISHE voltage, as presented in Supplementary Figure 7. The original data (V_d) shown in the figure (Supplementary Figure 7a) is detected at -1mA pulsed current with PZT polarized up. The jump around zero is ~0.22 mV. Firstly, the magnetic field (**H**) dependent background is Lorentzian peak function fitted as the green lines shown in the figure for H>0 and H<0 respectively (the coercive field range is ignored during fitting). The fitted background (V_f) is subtracted from the detected data, and the residuals (V_R) are further treated by adding the voltage signal at ±2 kOe, i.e., $V_{R,+H} = V_{d,+H} - V_{f,+H} + V_{+2kOe}$ and $V_{R,-H} = V_{d,-H} - V_{f,-H} + V_{-2kOe}$ for H > 0 and H < 0, respectively. The results are shown in Supplementary Figure 7b. The data are smoothed by the Savitzky-Golay filter as shown in Supplementary Figure 7c. A background voltage ~-0.53 mV in the whole magnetic field range attributed to the contact resistance and the resistance of Pt is further subtracted and the pure ISHE voltage is obtained in Supplementary Figure 7d. The ISHE voltage jump around zero field is thus 0.22±0.03 mV.

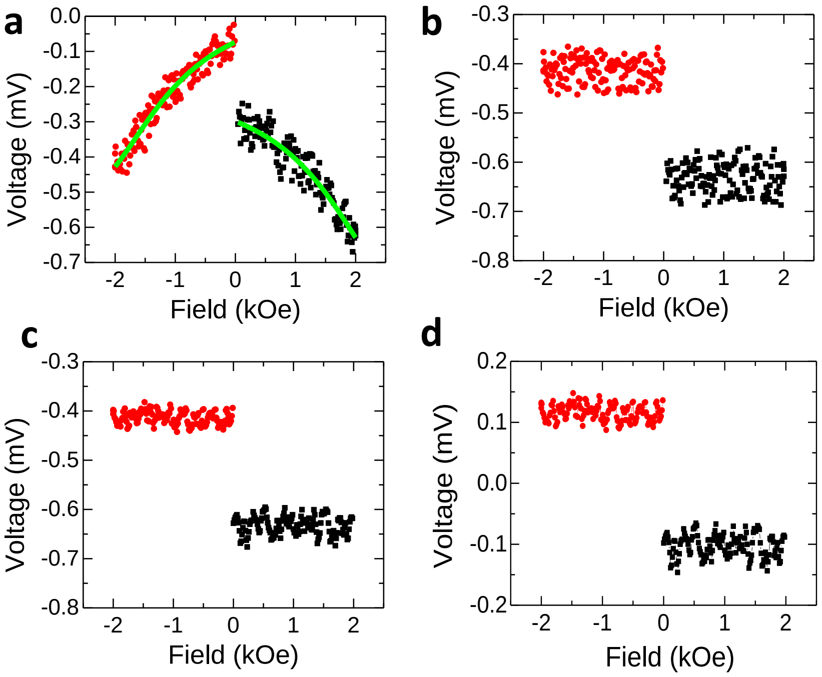

Supplementary Figure 7. Data processing: **a.** the experiment data (solid scatters) measured at -1 mA, and their Lorentzian peak function fitting (green lines) at +H and –H respectively; **b.** the results after subtraction of the fitted field-dependent part in **a**; **c.** the data after Savitzky-Golay filter process and **d.** the final t-pISHE signal.

Supplementary Note 7. Interfacial and bulk spin Hall angles

A semi-quantitative analysis of the voltage controlled interface spin Hall angle can be analyzed below. The spin current across the tunnel junction of LSMO/PZT/Pt is $\mathrm{J_s} = \mathrm{P_0 J_e}$ where P_0 denotes the spin polarization of the interface of LSMO (Supplementary Note 2) and J_e is the electrical current density across the barrier. The spin current will decay in the Pt layer. By using the boundary condition such that $\mathrm{J_s} = \mathrm{P_s J_e}$ at x = 0 and J_s = 0 at $x = d$ where d denotes the thickness of the Pt layer, and by using the diffusion equation, we have:

$$\mathrm{J_s}(x) = \mathrm{P_s J_e}\frac{\exp\left(\frac{d-x}{\lambda}\right)-\exp\left(\frac{d-x}{\lambda}\right)}{\exp\left(\frac{d}{\lambda}\right)-\exp\left(\frac{-d}{\lambda}\right)} \quad (7)$$

where λ is the spin diffusion length of Pt. The electric current density due to inverse spin Hall effect would be $\mathrm{J_{el}}(x) = \theta_0 \mathrm{J_s}(x)$ where θ_0 is the spin Hall angle of Pt. Integrating over x, we obtain the total electric current produced in the Pt layer,

$$\mathrm{I} = \mathrm{W}\int_0^d \mathrm{J_{el}}dx = \mathrm{P_s J_e W}\theta_0\lambda\tanh\left(\frac{d}{2\lambda}\right) \quad (8)$$

where W is the width of the Pt layer. If we model the interface contribution to the electric current by $\mathrm{I_{in}} = \mathrm{P_s J_e}\theta_{\mathrm{in}}\lambda_{\mathrm{in}}\mathrm{W}$, where λ_{in} is the spin Hall angle at the interface, $\lambda_{\mathrm{in}} = 0.1 - 0.2$ nm is the inelastic electron scattering length of electrons at the interface. The output voltage is:

$$V_{\mathrm{t-pISHE}} = \mathrm{P_s J_e WR}[\theta_{\mathrm{in}}\lambda_{\mathrm{in}} + \theta_0\lambda\tanh\left(\frac{d}{2\lambda}\right)] \quad (9)$$

where R is the resistance of Pt film. Note that R is also dependent on the thickness.

Ideally, by fitting the thickness dependence of the voltage for PZT polarization up and down, one could derive how θ_{in} varies when you change the PZT up to down (Fig.3c in the paper). The effective spin hall angle ($\theta_{\mathrm{SHE}}(\mathrm{net})$) can be determined as:

$$\theta_{\mathrm{SHE}}(\mathrm{net}) \equiv \frac{\mathrm{J_{el}}}{\mathrm{J_s}} = \frac{V_{\mathrm{t-pISHE}}\sigma}{\mathrm{J_e P_s L}} \quad (10)$$

since $\mathrm{J_{el}} = E\sigma = \frac{V_{\mathrm{t-pISHE}}}{L}\sigma$ and $\mathrm{J_s} = \mathrm{J_e P_s(V_e)}$ where σ ($\sigma = \frac{\mathrm{L}}{\mathrm{RW}d}$) and L are the conductivity and the length of the Pt strip. Using Supplementary Eq.(9), the $\theta_{\mathrm{SHE}}(\mathrm{net})$ is:

$$\theta_{\mathrm{SHE}}(\mathrm{net}) = \frac{\theta_{\mathrm{in}}\lambda_{\mathrm{in}}+\theta_0\lambda\tanh\left(\frac{d}{2\lambda}\right)}{d} \quad (11)$$

Supplementary Note 8. Interfacial complexity effects on Spin Hall Conductivity

To investigate the effect of interfacial complexity on spin Hall conductivity (SHC), we consider Ti-O terminated PZT substrate with interfacial Pt and Ti atoms interchanged, along with interfacial Pt and O atoms interchanged. The calculated SHC of monolayer Pt versus the number of valence electron under different cases of interfacial complexities when the ferroelectric polarization points out of the film surface (P-up) is shown in Supplementary Figure 8. For 1 Pt layer, we found SHC for interchanged Pt and Ti atom is similar to the sharp interface (near +1100 for both of the cases), while it decreases to negative value (~ -1500) near Fermi level once the interfacial Pt and O atoms interchanged. These results suggest the interfacial complexity play a key role in determining the sign of SHC in adsorbed Pt layers.

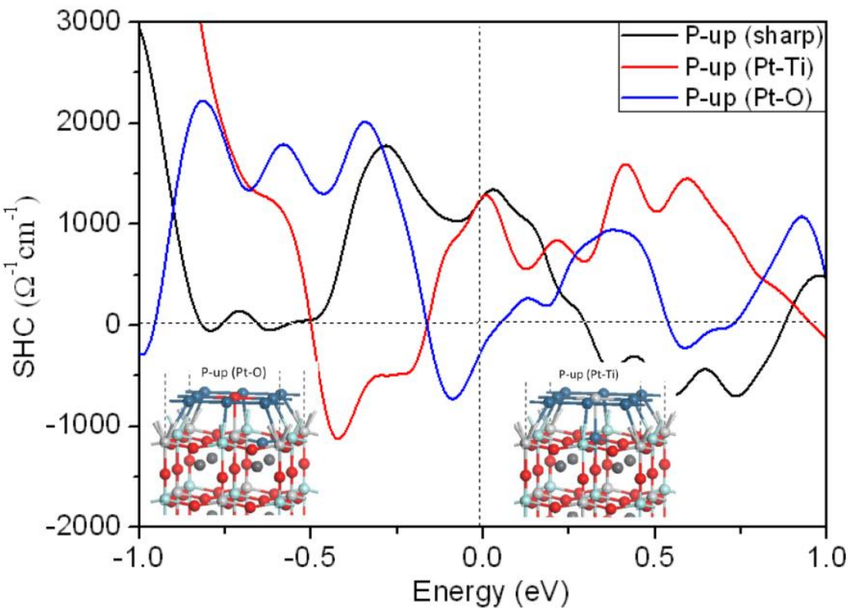

Supplementary Figure 8. Interfacial complexity effects on SHC of 1Pt layer on PZT which is polarized up. The black curve is for ideally sharp PZT/Pt interface, the red curve is for the interface with interchanged Pt and Ti atoms, and the blue one is for the interface with interchanged Pt and O atoms. At Fermi level, the SHC shows reverse for interchanged Pt and O atoms.

Supplementary Note 9. The Rashba effect at the PZT/Pt interface

To study the Rashba effect on the SHC in PZT/Pt, we take the first layer Pt on PZT as an example. To this end, the atomic models with one Pt layer on the PZT substrate with upward and downward ferroelectric polarizations (see Fig. 4a and Fig. 4b in the paper) are used here. Because the Pt layer gives rise to the spin Hall conductivity, we need to study the Rashba field sensed by it and thereby its Rashba bands. Due to the hybridization between the states of the Pt layer and PZT substrate, we obtain the bands of the Pt layer by projecting the wave functions of all bands only to the Pt layer (see the dots in Supplementary Figure 9). After determining the Rashba bands of the Pt layer, we can get the Rashba parameter α_R by fitting the Rashba bands in the vicinity of the $\mathbf{\Gamma}$ point to the formula $\Delta E = E_1(\mathbf{k}) - E_2(\mathbf{k}) = -2\alpha_{\mathrm{R}}\mathbf{k}$ where **k** is a vector in the reciprocal space and $E_1(\mathbf{k})$ and $E_2(\mathbf{k})$ are the DFT calculated energies of spin-up and spin-down channel bands. To get a reliable fitted Rashba parameter α_{R}, 25 points for the **k** are sampled in our fitting. Supplementary Figure 9 shows the DFT calculated band structures for both upward and downward ferroelectric polarizations of PZT. When the PZT polarization is upward, large spin-dependent shifts (highlighted by blue and red color) toward ±X direction can be found for a pair of Pt bands, suggesting the strong Rashba effect because of the interplay between the PZT electric field and Pt spin-orbit coupling (Rashba parameter is $\alpha_{\mathrm{R}}^{\uparrow} = -152\ \mathrm{meV}\cdot\mathrm{Å}$). When the PZT polarization is downward, a new pair of Rashba states are found in the PZT layer (black lines around Fermi level in Supplementary Figure 9b), and the spin-splitting state in the Pt layer disappears.

Assuming that the spin momentum time, τ, in the Pt layer is 5 fs and the thickness of the interface, d_{in}, is 0.2 nm, the derived inverse Rashba-Edelstein effect (IREE) length, $\lambda_{\mathrm{IREE}} = \alpha_{\mathrm{R}}^{\uparrow}/\hbar$, is 0.02 nm and the contribution from the IREE to an effective spin Hall angle is $\theta_{\mathrm{ESHE}}^{\mathrm{IREE}}=2\lambda_{\mathrm{IREE}}/d_{\mathrm{in}}=$ -0.18 for the upward PZT polarization. This value agrees with the estimated interfacial spin Hall angle in Eq. (2) that accounts for the negative sign of ISHE response. When the PZT polarization is inverted to the downward direction, the IREE is negligible. Thus the spin-to-charge conversion is merely determined by the positive bulk spin Hall angle, producing the positive ISHE response as PZT polarizes downward.

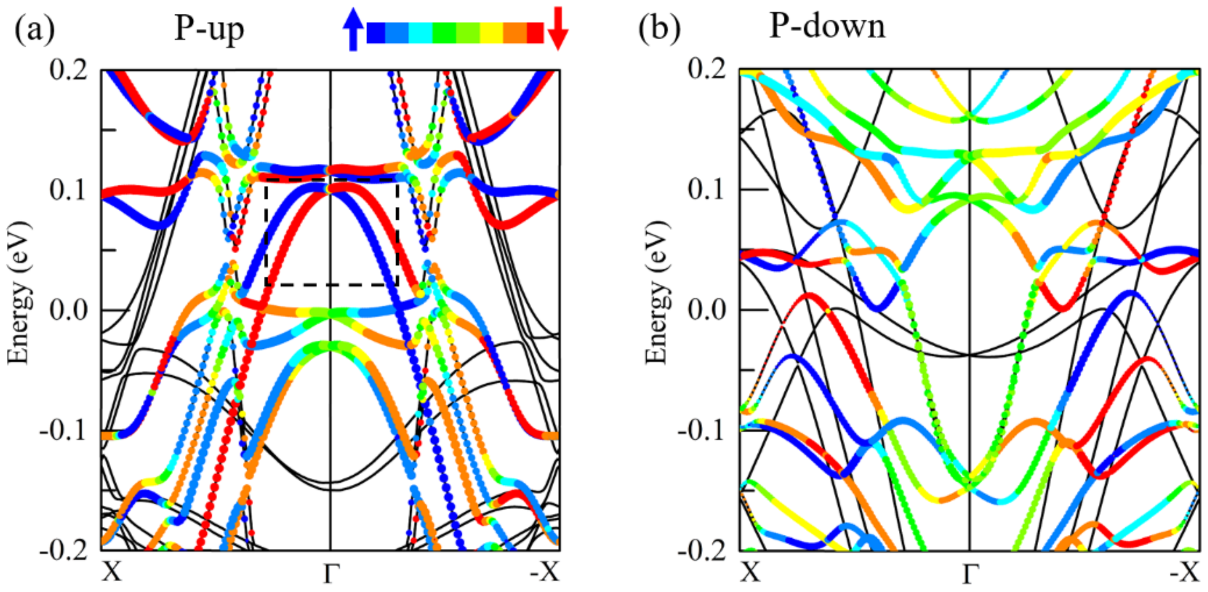

Supplementary Figure 9. DFT calculated band structures of one monolayer of Pt at the PZT surface with **a**. upward and **b.** downward ferroelectric polarization of PZT. The bands from the Pt layer are shown by dots in **a** and **b**. The Rashba bands from the Pt layer are highlighted by the black dashed lines in **a**. The color bar gives the spin projection weights.

References:

1. Kawasaki, M. et al. Atomic Control of the $SrTiO_3$ Crystal Surface. *Science (New York, N.Y.)* **266**, 1540-2 (1994).
2. Valenzuela, S.O., Monsma, D.J., Marcus, C.M., Narayanamurti, V. & Tinkham, M. Spin Polarized Tunneling at Finite Bias. *Physical Review Letters* **94**, 196601 (2005).
3. Liu, L., Chen, C.-T. & Sun, J.Z. Spin Hall effect tunnelling spectroscopy. *Nature Physics* **10**, 561-566 (2014).
4. Fang, M. et al. Nonvolatile Multilevel States in Multiferroic Tunnel Junctions. *Physical Review Applied* **12**, 044049 (2019).
5. Julliére, M. Tunneling between ferromagnetic films. *Physics Letters A* **54**, 225-226 (1975).
6. Bui, C.T. & Rivadulla, F. Anomalous and planar Nernst effects in thin films of the half-metallic ferromagnet $La_{2/3}Sr_{1/3}MnO_3$. *Physical Review B* **90**, 100403 (2014).
